# Dental implants placed on bone subjected to vertical alveolar distraction show the same performance as those placed on primitive bone

**DOI:** 10.4317/medoral.18545

**Published:** 2013-03-25

**Authors:** Mario Pérez-Sayáns, María A. León-Camacho, José M. Somoza-Martín, Beatriz Fernández-González, Silvia Blanes-Vázquez-Gundín, José M. Gándara-Rey, Abel García-García

**Affiliations:** 1 Oral Medicine, Oral Surgery and Implantology Unit. Faculty of Medicine and Dentistry. Institute of Sanitary Research of Santiago (IDIS); 2 Oral Medicine, Oral Surgery and Implantology Unit. Faculty of Medicine and Dentistry, Santiago de Compostela, Spain; 3 Oral Medicine, Oral Surgery and Implantology Unit. Faculty of Medicine and Dentistry. Institute of Sanitary Research of Santiago (IDIS). Santiago de Compostela, Spain

## Abstract

Introduction: Vertical osteogenic alveolar distraction (VOAD) allows for the augmentation of the alveolar ridge for the placement of dental implants in atrophic alveolar ridges. The goal of this paper is to assess long-term peri-implant bone resorption in implants placed on bones subjected to VOAD, comparing it with a group of patients who had implants placed directly on the alveolar bone without previous bone regeneration. Material and Methods: We conducted a follow-up study on 32 patients who were divided into two groups: The Distraction Group (14 patients), and the Distraction-Free Group (18 patients), who received a total of 100 implants. Peri-implant bone loss was measured by means of panoramic X-rays, at the time of loading and one year later, and in 35 implants of each group after 3 years of functional loading. Results: The peri-implant bone resorption (PBR) average observed in the Distraction Group at the time of prosthetic placement is higher (0.50±0.09 mm) than in the Distraction-Free Group (0.25±0.06 mm), showing statistically significant results (p=0.047). PBR levels 1 year after loading were the same for both groups (0.66 mm). At 3 years, they were higher in the Distraction Group (1.03 ± 0.22 mm vs. 0.68 ± 0.08 mm).

** Key words:**Bone resorption, alveolar distraction osteogenesis, dental implants.

## Introduction

Alveolar atrophy may limit the placement of dental implants, which has become a highly-effective therapeutic option for the rehabilitation of partially or totally edentulous maxillaries (1), as long as they are placed in the right conditions, thereby influencing the prognosis of implant-supported treatment (2,3). Vertical osteogenic alveolar distraction (VOAD) allows for the augmentation of the alveolar ridge to place dental implants in atrophic alveolar ridges. Compared to other surgical techniques proposed for this purpose, VOAD offers advantages such as not requiring a donor area, reduced resorption risk, placement of implants in a relatively short period of time and reduction of prosthetic height, decreasing crown-implant relation and ultimately improving treatment results (4).

Implant therapy effectiveness can be assessed by analyzing the maintenance of osseointegration when implants are subjected to long-term functional loading. This is verified by the study of peri-implant bone resorption, which stands out as one of the key factors within oral implantology success criteria (5-7). Although studies on implant placement on distracted alveolar bone are frequently published (8-10), few have shown its long-term reliability and those that have addressed this matter are even scarcer (10-12). Therefore we cannot know precisely if the alveolar bone that has been subjected to vertical bone distraction has the same performance as alveolar bone that has not undergone any type of regeneration when dental implants are placed.

The goals of this paper are to assess long-term peri-implant bone resorption around implants placed on bones that have been previously subjected to vertical osteogenic alveolar distraction , and to compare the bone resorption obtained in our study group with that observed in a group of patients who received implants on alveolar bone without any previous bone regeneration.

## Patient and Methods

We performed a follow-up study on 32 patients treated at the Master of Oral Medicine, Surgery and Implantology of the Faculty of Dentistry of the University of Santiago de Compostela, during a 3-year period. All patients required oral rehabilitation through an implant-supported treatment. In those cases in which we verified a future inadequate crown-implant relation (> 1) and/or an alveolar ridge with varying degrees of resorption and insufficient height to place implants with a proper length, we performed vertical alveolar osteogenic distraction to correct this defect, prior to implant placement.

The patients were divided into two groups. The first group (Distraction Group) was subjected to vertical alveolar osteogenic dis-traction surgery before implant placement. And the second group (Distraction-Free Group) received implant surgery without any bone regeneration before or during the procedure.

-Distraction Group 

The Distraction Group was composed by 14 patients (8 women and 6 men) with a mean age of 43.29 years (age range 22 to 58 years), who were consecutively subjected to 21 vertical osteogenic alveolar distraction , 19 located in the mandible and 2 in the superior maxilla ( Table 1).

**Table 1:**
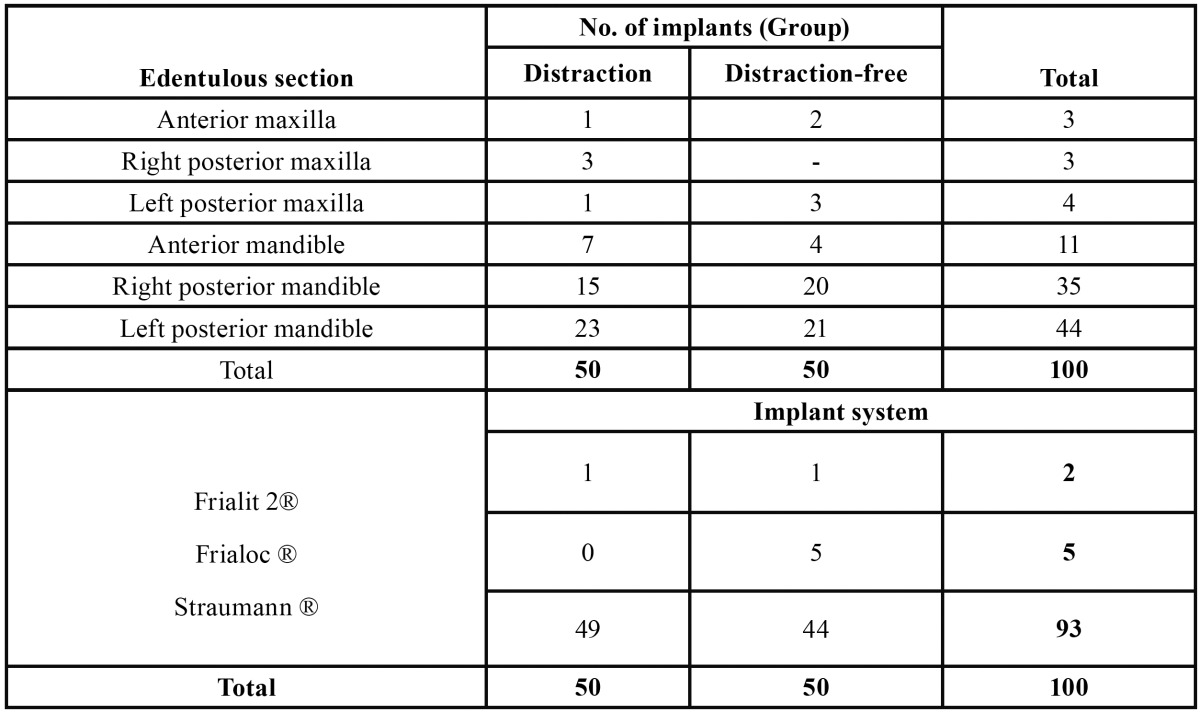
System and number of implants placed per edentulous section in the distraction and the distraction-free group.

-Distraction-Free Group 

The Distraction-Free Group was formed by 18 patients (10 women and 8 men) with a mean age of 50.78 years (range 38 to 67 years), who received a total of 50 implants in 25 wholly or partly edentulous alveolar ridges, 23 in the mandible and 2 in the maxilla ( Table 1).

-Distraction protocol

VOAD surgeries were performed by the same surgeon, using 2 different types of distracters: In 18 cases we used the semi-rigid intraosseous distracter LEAD System® (Leibinger, Kalamazo, USA), 2 in the upper maxilla and 16 in the mandible. In 8 of these osteodistractions we placed 2 distracters per segment. The other 3 alveolar distractions were made using a rigid alveolar juxta-osseous distracter Modus MDO System® (Medartis, Basel, Switzerland), all located in the mandible.

After a 7-day latency period, distraction was commenced at a rate of 1 mm/24 hours in the maxilla or 0.5 mm/12 hours in the mandible. After a consolidation period of 12 weeks, the distracter was removed and the implants were placed in a single surgical intervention. After another 12 weeks of osseointegration, the implants were loaded. Patients were clinically monitored during all stages of the distraction protocol. An orthopantomography was done 24 hours after surgery; a second one was taken at the end of the distraction period and a third immediately before the implant surgery, in order to address any complications that might arise and to reassess implant planning.

-Implant placement and prosthetic rehabilitation 

The implant surgery was performed following the directions and recommendations of the implant system. We inserted a total of 50 implants on the 21 distracted segments: 44 Straumann®, 5 Frialoc® (Frialit, Freiburg, Germany) and 1 Frialit 2® (Friadent, Mannheim, Germany), 45 placed on the mandible and 5 in the superior maxilla. We placed a total of 50 implants in the 25 eden-tulous segments of the Distraction-Free Group: 49 Straumann® (Waldenburg, Switzerland) and 1 Frialit 2® (Friadent, Mannheim, Germany), 45 placed on the mandible and 5 in the superior maxilla ( Table 1).

In the Distraction Group, implant diameter oscillated between 3.3 mm and 4.8 mm, and their length ranged between 8 mm and 13 mm. In the Distraction-Free Group, implants varied from 3.3 mm to 4.8 mm in diameter and 8 mm to 15 mm in length.

During implant surgery the Distraction Group showed bone defects (7 dehiscences and 5 fenestrations), which were regenerated with xenogeneic and alloplastic materials. In the Distraction-Free Group was no bone regeneration was performed.

Prosthetic rehabilitation was carried out in both groups, following the directions proposed by the implant system and verifying their clinical setting by taking panoramic X-rays from the time of insertion of the prosthesis.

-Evaluation of peri-implant bone resorption

**Figure 1:**
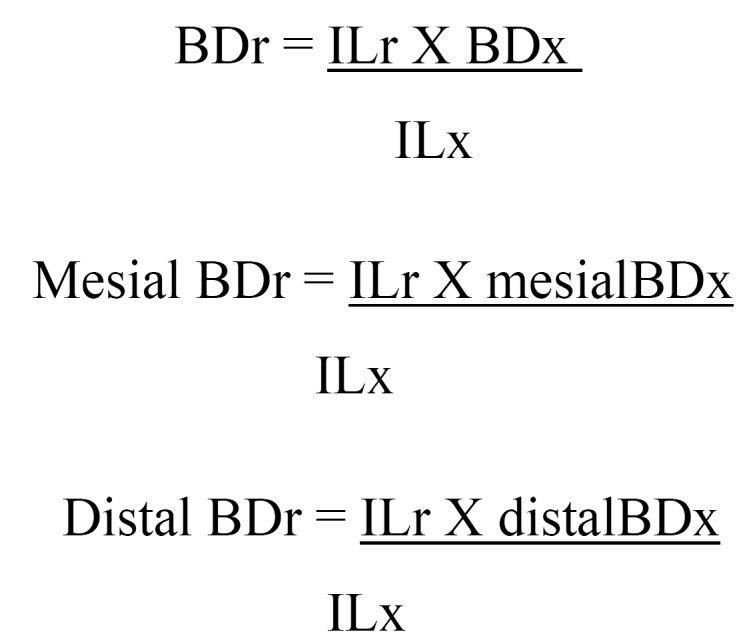
Evaluation of peri-implant bone resorption.

Peri-implant resorption was evaluated using panoramic X-rays obtained on the day of implant loading, which were repeated 1 and 3 years later. Two measurements were obtained: 1) radiologic bone deficit (BDx), the distance from the neck of the implant to the bone crest, measured both mesial and distal to the implant, and 2) radiologic implant length (ILx), the distance from the neck of the implant to its apical extreme. Actual implant length (ILr), the known length of the intraosseous part of the implant plus neck length (Straumann standard _ 2.8 mm; Straumann plus _ 1.8 mm; Frialoc _ 2.5 mm; Frialit _0.4mm), was also obtained. Real bone deficit (BDr) was calculated both mesially and distally as follows. (Fig.1)

Finally, vertical peri-implant resorption values were calculated as follows:

Mesial resorption = (mBDr at 1 and 3 years after loading) – (mBDr at loading)

Distal resorption = (dBDr at 1 and 3 years after loading) – (dBDr at loading)

Mean resorption = (mesial resorption + distal resorption) / 2

-Statistical analysis 

In each group we related age, sex, diameter and length of implants with peri-implant bone resorption. Meanwhile, these results were compared within each group. The data from this study was statistically analyzed using the program SPSS 16.0 for Windows XP. The results were expressed as mean ± standard error of the mean. To compare peri-implant bone resorption between the time of loading, 1 year and 3 years, we used the Wilcoxon signed-rank test for paired data. To compare bone resorption between the Distraction and Distraction-Free Groups, we used the non-parametric Mann-Whitney test. To find the relationship between quan-titative variables, we used the Spearman’s rank correlation coefficient. Statistical significance was set at P<0.05.

## Results

-Study of peri-implant bone loss

In the Distraction group, there were no statistically significant differences in peri-implant bone resorption between the time of loading and one year (p=0.512) and between the time of loading and 3 years after functional loading (p=0.457) .

In the Distraction-Free Group there were statistically significant differences between the time of loading and one year (p=0.000) and between time of loading and 3 years later (p=0.003).

The peri-implant bone resorption average observed in the Distraction group at the time of prosthetic placement was higher (0.50±0.09 mm) than in the Distraction-Free group (0.25±0.06 mm). These results were statistically significant (p=0.047).

**Figure 2:**
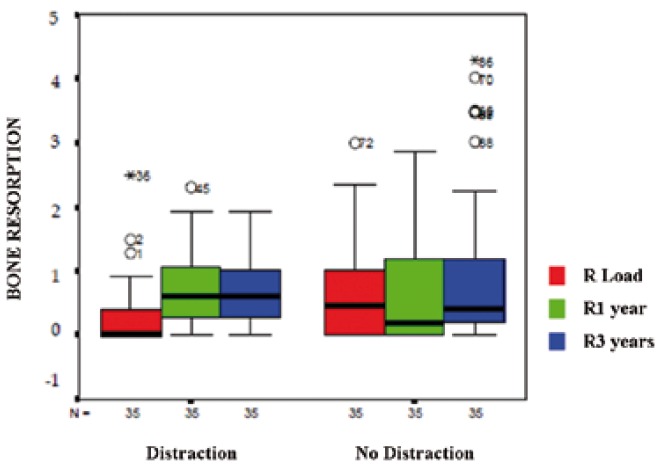
Comparison of peri-implant bone resorption between the Distraction and the Distraction-Free group, at the time of loading, after 1 year and after 3 years.

Taking the loading values as a reference for comparing the resorption at 1 and 3 years in both groups, we found evidence that levels of bone loss 1 year after loading was equal in the Distraction Group and in the Distraction-Free Group. At 3 years, these were higher in the Distraction Group, but did not reach statistical significance ( Table 2), (Fig. 2).

**Table 2:**
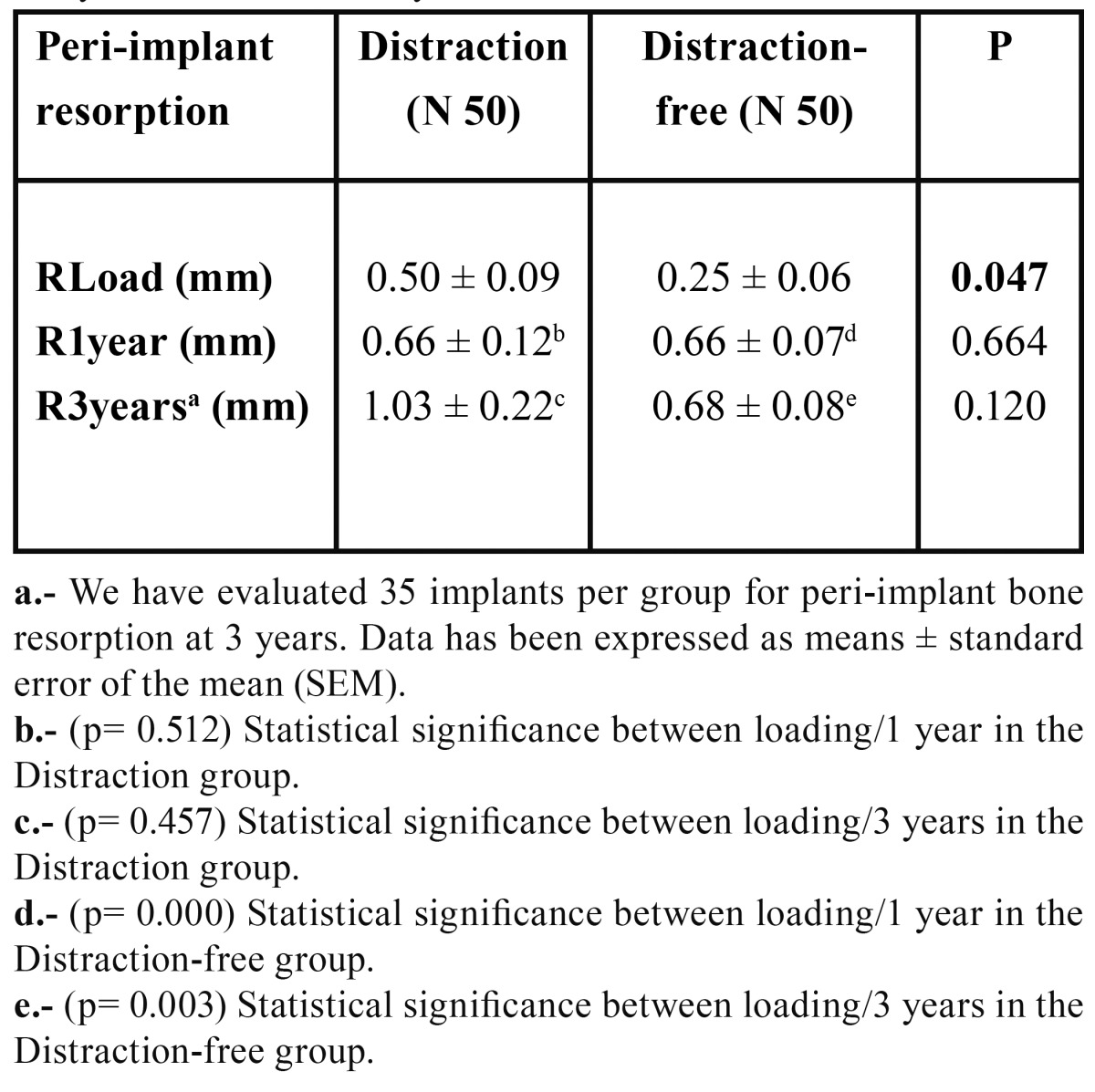
Comparative values and statistical significance between peri-implant bone resorption in both groups at the time of implant, one year later and after 3 years of functional load.

-Peri-implant bone resorption in relation to other variables

-Sex / Peri-implant bone resorption 

**Figure 3:**
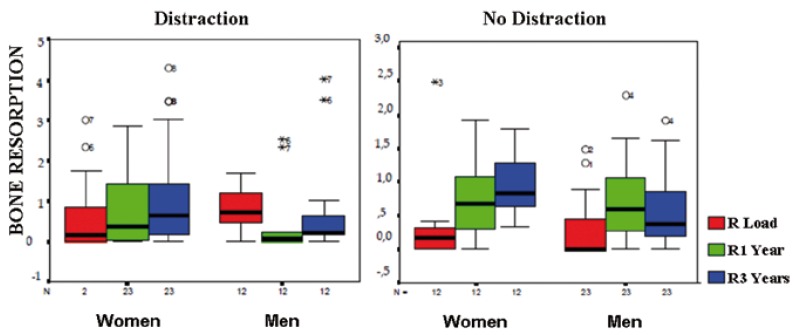
Relationship between sex and peri-implant bone resorption at the time of loading, and at 1 and 3 years after functional loading, in the Distraction and Distraction-Free groups.

By relating the sex of both groups with peri-implant bone resorption, we observed that the Distraction Group showed a greater average of resorption in men (0.54 ± 0.11 mm) at the time of loading. At 1 and 3 years after loading, bone loss was greater in women, but these results did not reach statistical significance. Whereas, in the Distraction-Free Group mean resorption at the time of loading was higher in men (0.27 ± 0.08 mm) than in women (0.23 ± 0.10 mm). 1 year after loading, the average resorption was equal for both sexes, however, we observed no statistically significant differences. At 3 years, the average resorption was higher in women, showing statistical significance: p=0.014 (Fig.3).

-Age / Peri-implant bone resorption

**Figure 4:**
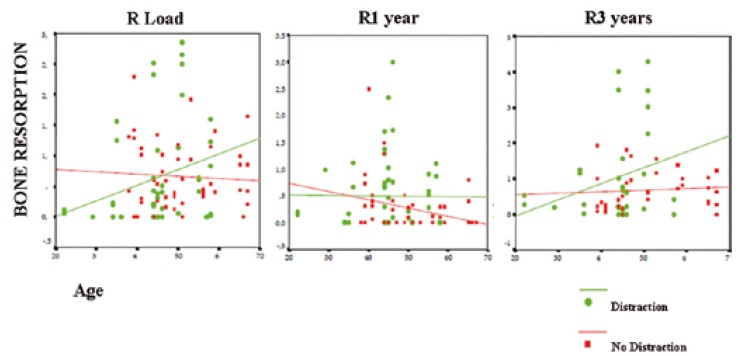
Relationship between age and peri-implant bone resorption at the time of loading, and at 1 and 3 years after functional loading, in the Distraction and Distraction-Free groups.

In the Distraction Group, bone loss at the time of functional loading was inversely proportional to age (R=-0.76). That is, higher resorption levels were observed in younger patients, although these results were not statistically significant (p=0.600). However, one year later (R=0.306) and 3 years after loading (R=0.168), this correlation was reversed, showing an increase of bone loss in older patients, although the only statistically significant difference was observed after one year of functional loading (p=0.030). In the Distraction-Free Group the mean peri-implant bone resorption was higher in younger patients at the time of prosthetic inser-tion (R=-0332), showing a statistically significant correlation (p=0.019). 1 year after loading, this proportion was sustained (R=-0.28), showing no statistical significance (p=0.847). However, this proportion changed at 3 years after loading, where bone loss increased in older patients (R=0.244), but these values did not reach statistical significance (p=0.157). These results are expressed in figure 4 through scatter plots.

-Implant Diameter / Peri-implant bone resorption 

At the time of loading, the Distraction Group showed greater bone loss around small diameter implants (R=-0256), but this correlation was not statistically significant (p=0.073). One year later (R=0.191) and 3 years later (R=0.136) bone resorption increased and was higher in larger diameter implants. However, none of these results were statistically significant. In the Distraction-Free Group, at the time of prosthetic insertion, smaller diameter implants showed greater resorption (R=-0395), thus showing statistical significance (p=0.005). One year later (R=0.159) and 3 years later (R=0.065), larger diameter implants showed increased peri-implant bone resorption. However, these values were not statistically significant.

-Length / Peri-implant bone resorption 

In the Distraction Group, shorter implants showed greater peri-implant resorption at the time of prosthetic insertion (R=-0055), a proportion that was sustained one year after loading (R=-0113). These results showed no statistical significance. At 3 years (R=0.111), this relationship was reversed, showing higher bone resorption in longer implants. In the Distraction-Free Group, at the time of loading, peri-implant bone resorption was directly proportional to the length of the implants (R=0.109), and did not show statistical significance (p=0.451). Whereas, a year after loading, shorter implants showed higher resorption (R=-0323), and these results were statistically significant (p=0.02). However, at 3 years from implant loading, this proportion changed, showing a higher bone loss around longer implants (R=0.131), but without statistical significance (p=0.453).

## Discussion

The replacement of conventional dental prosthesis for implant-supported treatments occurs more frequently and has been consolidated thanks to the emergence of studies that prove its long-term effectiveness (13). Difficulties arise when the conditions of the patient’s oral cavity are not appropriate for this treatment. Insufficient height of the residual alveolar ridges often represents a major constraint for the placement of implants. Thus, alveolar bone distraction recently appeared as an alternative surgical procedure used for the reconstruction of this type of alveolar ridges, prior to implant treatment.

In this paper, we performed a follow-up study on 32 patients, of which 14 (Distraction Group) were subjected to vertical osteogenic alveolar distraction, prior to implant surgery. 50 implants were analyzed from the time of insertion of the prosthesis until 1 year later; while 35 implants were monitored at 3 years of their functional loading. In the 18 remaining patients (Distraction-Free Group), we placed the same number of implants and we performed a follow-up study as in the Distraction Group. Both groups showed similar characteristics in terms of age, gender, edentulous area, number, location, implant system and follow-up time, being as homogeneous as possible thus allowing us to compare them on equal terms (avoiding a statistical bias).

During the Distraction Group implant surgery we observed bone defects in 50% of cases once the implants had been inserted, basically in the form of dehiscences and fenestrations. Saulacic et al. (14,15) published two separate studies in 2007, revealing 51.16% and 58.62% of bone defects after implant placement. Other authors referred to the need for bone regeneration procedures because of the bone defects found when removing the distracter and placing dental implants (16,17).

The number of implants evaluated and the follow-up period of our Distraction Group was consistent with other previously published studies, which also evaluated peri-implant bone loss under these same circumstances and in which they employed a number of implants and study time similar to our research study (10,18-23). Panoramic X-rays have been the main tool for analyzing peri-implant bone resorption in both groups as well as in other studies (10,12,23,24). Panoramic X-rays provide sufficient reliability in vertical measurements, as long as the magnification factor is well determined or the distortion has been included in the measurements, as in this research project. After assessing our preliminary results, we observed that the differences found in peri-implant bone resorption between the 2 and 3 year follow-up sessions were virtually imperceptible radiographically and statistically, which, coupled with the fact of having to expose patients to more radiation without justification, was not offset by the results. For this reason, we decided distribute radiographic tests in the following way, we performed an initial X-ray at the time of loading, followed by a second one 1 year later and another one 3 years later.

The differences of the values of peri-implant bone resorption found in the Distraction Group, from the time of prosthetic insertion to the first year after loading (0.66 ± 0.12 mm) and 3 years later (1.03 ± 0.22 mm) were not statistically significant. However, when comparing these results with the Distraction-Free Group, we found a statistically significant difference at the time of im-plant loading (p=0.047), revealing a greater bone loss in the Distraction Group. It is possible that these results are related to the fact that 50% of our VOAD cases showed bone defects when inserting implants (mainly dehiscences). Although we performed bone regeneration procedures to remedy the most important bone defects, it is possible that full bone regeneration had not taken place at the time of placing the prosthesis. Subsequent results further support this analysis since 1 year after loading both groups showed similar levels of resorption. At 3 years, the Distraction Group exceeded the average of bone loss, but these results were not statistically significant.

The peri-implant bone loss observed in the implants placed on distracted alveolar bone in our study was similar to what had been described in previous research regarding implants placed on native bone (13,22). Furthermore, some of these publications show higher average bone loss than our results (25-27). Some of the patients of the Distraction Group did not show peri-implant resorp-tion. However, in contrast, we observed bone gain, which is consistent with Behneke et al. (13), who also reported bone gain in 24% of studied implants.

Very few papers have evaluated long-term bone resorption around implants placed on alveolar bone that had previously under-gone VOAD. In a preliminary study, Chiapasco et al. (10) reported an average of peri-implant bone loss of 1.3 mm, 1 year after loading they reported it in 26 implants using 2 different systems. Resorption was similar for both types of implants and their cu-mulative success rate reached 100%. Polo et al. (22) observed an average resorption of 1.9 mm after one year of functional load-ing, in a total of 34 implants placed in the posterior mandible and reconstructed by vertical alveolar bone distraction. The survival rate was 94.1%. Perez-Sayans et al. (23) recorded an average bone resorption of 0.60 mm and 0.68 mm in the mesial and distal surfaces in 37 implants, with a survival rate of 100%. In our Distraction Group, with the same loading time, we observed a sig-nificantly lower bone resorption average (0.66 ± 0.12 mm) in 50 implants.

Chiapasco and his research team performed two consecutive studies in more implants and for a longer period of time. In one of these studies (11) they analyzed 138 implants over a period ranging from 1 to 4 years. Average bone resorption was 0.8 mm after one year of functional loading. In subsequent years, the average bone loss was 0.1 mm in the second and third year, and 0.2 mm 4 years after loading, significantly decreasing resorption levels in the long term. The survival rate was 100%, whereas success rate reached 94.2%. The second paper presented by the same research group (20) consisted of a comparative study between the GBR and VOAD. The mean bone loss in 34 implants was 1.3 mm at 1 year after loading, followed by 0.1 mm and 0.2 mm in the second and third year after prosthetic rehabilitation. In the latest studies published by this author on VOAD (21), he compares its effectiveness against that of onlay grafts. The VOAD Group received 21 implants, showing an average bone resorption of 0.9 mm 1 year after functional loading, and 0.1 mm 2 and 3 years later, followed by 0.3 mm at 4 years. In these 3 papers, bone resorption 1 year after functional loading was higher than in our study. However, in the subsequent years, bone loss is lower, which is an improvement in comparison to our results.

According to the success criteria proposed by Albrekttson et al. in 1986 (7), we can confirm that the implants in our study group showed a survival rate of 100%. By relating the peri-implant bone loss in the study groups with other independent variables such as age, sex, implant diameter and length, we have registered inconclusive results. The great controversy between the results found in the literature is also evident, possibly because implant-supported rehabilitation affects more than one isolated parameter (4,28-30). Therefore, one might conclude that a number of factors influence and interfere in the success of this type of treatments, such as the size of the alveolar ridge, the direction of occlusal forces, the size of the prosthesis and the number of implants sustaining it, the crown-implant ratio, peri-implant tissue health, oral hygiene and harmful habits, most notably smoking, and a strict maintenance phase.
